# The journey of Radiology in Ethiopia

**DOI:** 10.4314/ejhs.v32i1.11S

**Published:** 2022-10

**Authors:** Tesfaye Kebede, Daniel Zewdeneh, Asfaw Atnafu, Daniel Admassie, Getachew Asefa, Yocabel Gorfu, Asefa Getachew

**Affiliations:** Department of Radiology, College of Health Sciences, Addis Ababa University

**Keywords:** Radiology, Journey, Ethiopia

## Abstract

The practice of radiology began after the invention of X-rays in 1895 which then spread to different parts of the world. There is no documentation on how and when the x-ray was introduced to the Ethiopian medical practice. However, radiology as a profession was in place for the last four decades. Similar with the experience in other countries, the history of progress in the field of radiology in the Ethiopian setup is related directly to technological advances that occurred during the past few decades. Radiography was the main modality used in the first two decades. In the early years of the initiation of radiology training, only radiographs and ultrasounds were available for training and service. In the subsequent years, modern cross-sectional imaging equipment was introduced. This was mainly accomplished with the involvement of the private institutions which played a significant role. So far, there are more than 300 practicing radiologists as diagnosticians. Recently, also radiologic interventions were also introduced with the commencement of subspecialty training.

## Introduction

There is no recorded account or historical evidence as to when and how radiology was formally introduced into Ethiopia. On the world stage, though, medical information from historical archives shows that soon after Roentgen discovered X-Rays in 1895; the first ever medical use of X rays was recorded in Napoli, Italy in 1897. It was officially used in hospitals, ironically enough, for wounded soldiers who returned from the battle of Adowa, Ethiopia (1) Although there is no documentation regarding the history of medical service or education (2), it is probably assumed that x-ray could have found its way to Ethiopia during the second Italian invasion and occupation as part of their medical service for their army and civilian contingent (3). This seemed a very likely possibility as a lot of infrastructure and other developmental ventures were indeed undertaken during this time. This was also further corroborated by information through word of mouth by our forebears who lived at the time.

Eventually in the late 40s, 50s, and well into the 60s; a significant number of European doctors were already practicing medicine. X-ray and direct fluoroscopy were in wide use in Addis Ababa, and other towns like Harar, Asmara, and Jimma. Some of the pioneer radiologists in our country had even seen and used these technologies in the early years of their medical and radiology practice which was in the 80s when they started to practice as radiologists. Soon after the end of the Italian war, especially after the establishment of the Ministry of Health in 1948; the government of Ethiopia undertook an earnest modernization of medical service in Ethiopian hospitals (3,4). Since then, and in the last three decades, radiology has come a long way. Even if the public health institutions have enormous challenges in introducing modern imaging equipment and assuring sustainable services due to many reasons, especially the long bureaucratic lines and obstacles, the private health institutions were instrumental in filling the gap and contributing to the development of radiology in Ethiopia. This article aims to briefly address its historical journey.

## Methods

This is a retrospective study analyzing the journey of radiology in Ethiopia and its current status. The study was conducted in Addis Ababa, Ethiopia. Data were collected from the archives of the first radiology department of Addis Ababa University, through interviews made with the pioneer radiologists and founders of the Ethiopian radiology training, and from published works about radiology in Ethiopia. Data were analyzed and structured in chronological order. Gender distribution of radiology training was obtained from all radiology schools by using a structured questionnaire with the year of graduation and gender of the graduates.

## Findings

**Figure 1:**
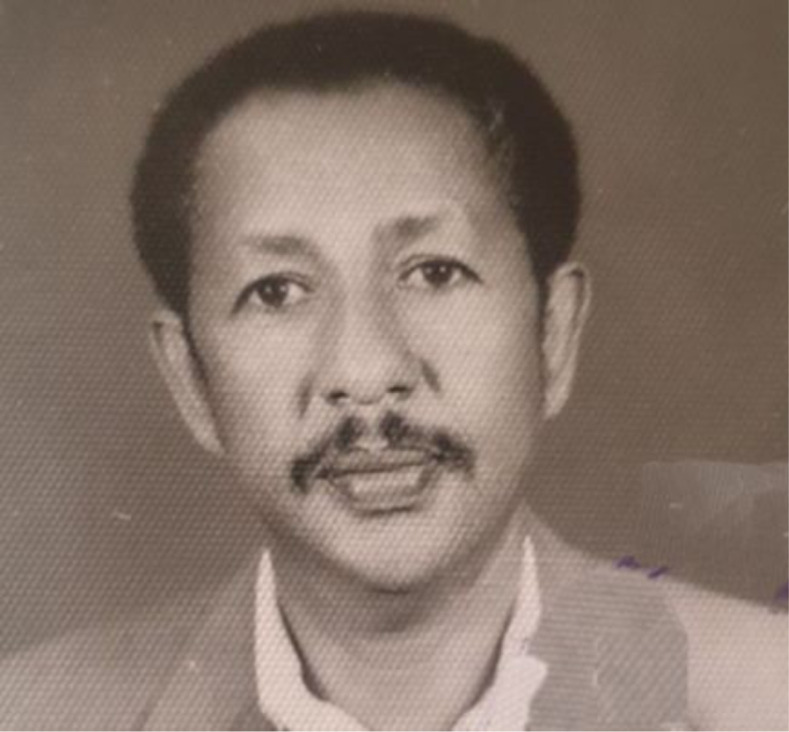
Dr. Tsegaye Desta, one of the first two Ethiopian radiologists who were trained oversea.

**The pre-radiology training era**: This period spans roughly 7 decades at which time x-ray and fluoroscopy were widely in use and were obviously run by medical doctors, mostly expatriates from Europe who came to practice medicine or as part of missionary contingents in various towns of the country. In the 1970s, after the Derg came to power, doctors for Eastern Europe and the Soviet Union's Warsow Block took over en masse. These contingents included qualified radiologists who started to run the radiology service well up to the early 90s. By this time, radiographers were already being trained and replacing unqualified health personnel working in radiology in Ethiopia. Roughly, towards the end of the 80s, and for the first time in the country's history, the first two qualified Ethiopian radiologists trained overseas started proper radiology practice, namely, Dr. Mekbeb T/Mariam of Armed Forces Hospital, and Dr. Tsegaye Desta of Tikur Anbessa Specialized Teaching Hospital (Fig. 1). Radiology service by then was fairly advanced in these hospitals where general radiography, conventional tomography, and various contrast fluoroscopic investigations such as lymphovascular, gastrointestinal, genitourinary, and myelography were performed.

**Figure 2:**
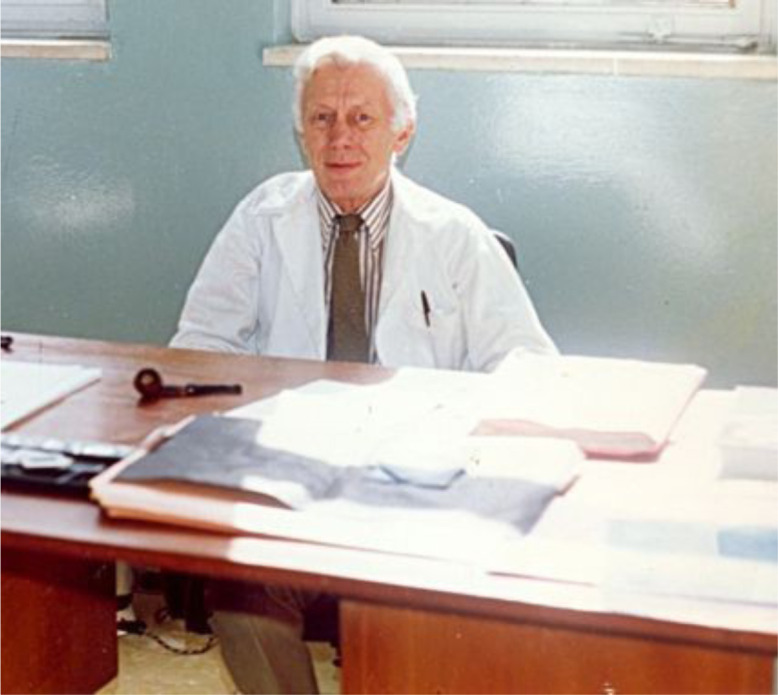
Professor Lesley Robert Whitaker, a British expatriate who started radiology training in Ethiopia.

**The Early Years of Radiology Training and the First Generation of Radiologists**: In the middle of the 80s, the Faculty of Medicine of Addis Ababa University began an earnest effort to launch graduate medical training. As part of this venture, and through the support of the British Overseas Development Agency (ODA): A British expatriate by the name of Professor Lesley Robert Whitaker (who had a background history of launching such programs in Kuwait, Seychelles, and Kenya) (fig. 2), was invited to start the graduate radiology residency program. After roughly two years of preparatory work, the program started with an intake of six trainees in 1987.

From then on, the program had remained steadfast till now despite challenges over the years. An article published in the South African Journal of Radiology in 1998 by Steve Beningfield, a visiting radiology professor from the University of Cape Town, described those early years of radiology training and service at TASH and the country at large; in a peculiar manner where he showed how the radiology training was making a difference in the quality of tertiary medical care despite challenges in terms of high end diagnostic equipment availability and other resource shortages with serious limitations similar to other African countries except for Egypt, South Africa, and surprisingly enough, Kenya, Nigeria, and Zimbabwe (4,5).

The graduate radiology program, under the tutelage of Professor Lesley R Whitaker, and through the aid from ODA, continued at a steady pace and enrolled two more resident batches back-to-back, right after the first intake. He stayed throughout the completion of the course in those three batches before he officially retired, leaving the program in the able hands of the late Dr. Tsegaye Desta, (fig. 1) (one of the first two Ethiopian radiologists trained overseas), and three of the newly graduated radiologists (Drs. Asfaw Atnafu, Getachew Assefa, and Daniel Admassie-who are still active in service and attained full professorship over the years of their tenure). Two more radiologists from the 3^rd^ batch were added, namely, Drs. Daniel Feleke, and Daniel Zewdneh (who has recently been promoted to full professor). To date, this graduate program has produced 265 graduates of which 60 (23%) are female radiologists (6).

**The Role of the Graduate Program in the Foundation of the Radiology Society of Ethiopia, (RSE)**: The year 1994 G.C. could be considered as a watershed period where the program had already produced 5 batches of radiologists totaling 20 in number and achieving the critical mass for a concerted effort in founding the Society. These graduates together with the few Ethiopian radiologists trained in Europe as well as a sizable expatriate contingent from Eastern Europe in practice at the time; gathered in the same year and launched the first inaugural conference of the Society's official formation. To date, the Society is robustly thriving with a vibrant future with more than 300 active members practicing in all corners of the nation and abroad.

**The first 20 years of training**: As shown in Table 1, the graduate program served as the only source of training for a little more than two decades in the country up to 2011 and produced the critical manpower, the number needed, to initiate similar programs in other universities across the nation and hence paved the way for expanding radiology.

**Table 1:** Radiology graduates from 1990 – 2012 from TASH and the succeeding years with the addition of five universities

Year	Male	Female	Total
1990	5	0	5
1991	3	0	3
1992	5	0	5
1993	2	2	4
1994	3	1	4
1995	0	0	0
1996	0	0	0
1997	3	1	4
1998	1	4	5
1999	1	3	4
2000	3	0	3
2001	3	1	4
2002	4	1	5
2003	5	0	5
2004	6	2	8
2005	6	1	7
2006	8	1	9
2007	7	3	10
2008	15	2	17
2009	7	2	9
2010	9	3	12
2011	6	4	10
**Subtotal**	**103**	**29**	**132**
**Succeeding years of Radiology**			
2012+Gondar	7	2	9
2013	4	1	5
2014	5	0	5
2015	16	6	22
2016	9	3	12
2017+SPHMMC	9	7	16
2018+ Mekelle	35	8	43
2019	41	9	50
2020+ Bahrdar	54	14	68
2021	45	22	67

Total	327	103	430

**Succeeding years of radiology**: In 2012, Gondar University which launched its program around 2009, graduated its first batch. Saint Paul Millenium Medical School joined the rank in 2017, followed by Mekelle University in 2018 and Bahrdar University in 2020 as shown in table 1. The number of radiologists produced since 2012 is inclusive of all five radiology programs. The above values in the table show that, since the launching of the program in the other institutes, there has been a 3-fold increase in the number of graduates of both genders. This is a very significant contribution to the professional workforce for tertiary healthcare service providers in the country. However, there is still room for improvement in terms of gender equity with only 102 (23.5 %) female radiologists out of a total of 433. As a pioneer program, the Department of Radiology of TASH, AAU, has achieved tremendously over the years with its graduates providing service throughout the country and laying down the foundation for graduate radiology training programs in other universities nationwide.

**Pioneer Female Radiologists and Gender Distribution of Radiologists in Ethiopia**: The program started enrolling female residents in the fourth batch (1990). Two young doctors by the name of Drs. Misrak Tilahun and Mekdes Lemma became the first female radiologists in Ethiopia. From here on, there was a steady and successive enrollment of female radiology graduates including one expatriate from Yemen but still, the male to female ratio is 4:1.

**Emerging New Radiology Schools**: The last ten years have shown a very encouraging trend in higher education, and as a result, graduate radiology training has sprouted in many universities where graduates from AAU have started offshoots of the program. Currently, there are four active programs (Millenium Medical School, Gondar University, Mekelle University, Bahrdar University), and one recently launched at Adama University. Jimma University was running the program but interrupted after graduating the first batches. These new programs are doing well, expanding their activities and making a difference in the overall quality of healthcare. There are promising signs that other universities in the country will follow suit.

**Figure 3:**
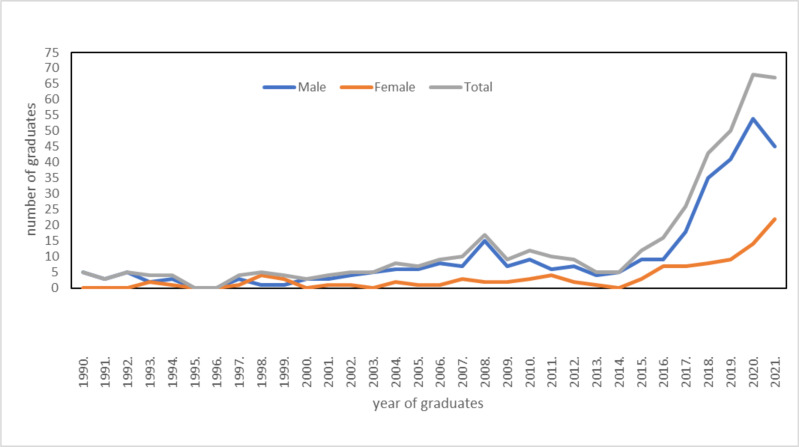
Trends of radiology graduates' profiles by year.

**Trends in Radiology Residency Training**: There has been a steady rise in the number of young doctors enrolling for radiology residency over the years with an equally encouraging rise in the number of female doctors (figure 3). This is probably because of the rapid advances in imaging technology and the meaningful influence on the improved tertiary healthcare providers nationwide. Even if the expansion of the postgraduate program started in the year 2003, there was no significant change except for a slight increase in the number of radiology graduates a few years after the launching of the expansion which happened in the existing radiology training program at AAU. Despite the fact that a new training program has started at Gondar, its intake capacity was too small to change the graduate numbers until the three new radiology training programs started in 2017 and afterward which injected a large number of professionals into the system (fig. 3).

**Challenges in Radiology Training Programs/Radiology Resources**: Radiology in Ethiopia has made a huge step forward in terms of professional manpower development and technology advancement over the years, albeit rather slowly; however, numerous challenges affect steady progress. The most outstanding setback, in the authors' view, is the inherent resource-intensive nature of radiology. In a third-world economic background like ours, where there are more national health needs that require stringent priority settings in the face of meager government fiscal allocation and where there is no policy for additional revenue generation: Advancing the pace of radiology becomes an insurmountable task. In addition to this, the lack of proper equipment acquisition and maintenance policy seriously affects the quality of training and healthcare service, to say the least. There is a lot of room for improvement in these two important spheres of concern and should be duly addressed by all stakeholders and at policy-making levels.

**Role of the private sector**: The private health institutions and diagnostic service providers in Ethiopia played a significant role in the history of radiology in Ethiopia. In addition to expanding the radiology services in the country and creating demand for professional manpower, they also pioneered in introducing modern imaging equipment to the radiology practice. Due to the less bureaucratic process in private health institutions and less equipment downtime, unlike the public ones, there is relatively sustainable service. Some of the private institutions were also used as teaching sites for radiology training.

**Academic collaborations and Fellowship in Radiology**: The three academic collaborations which impacted the practice of radiology in two of the radiology training institutions are as follows The case from AAU

**Figure 4:**
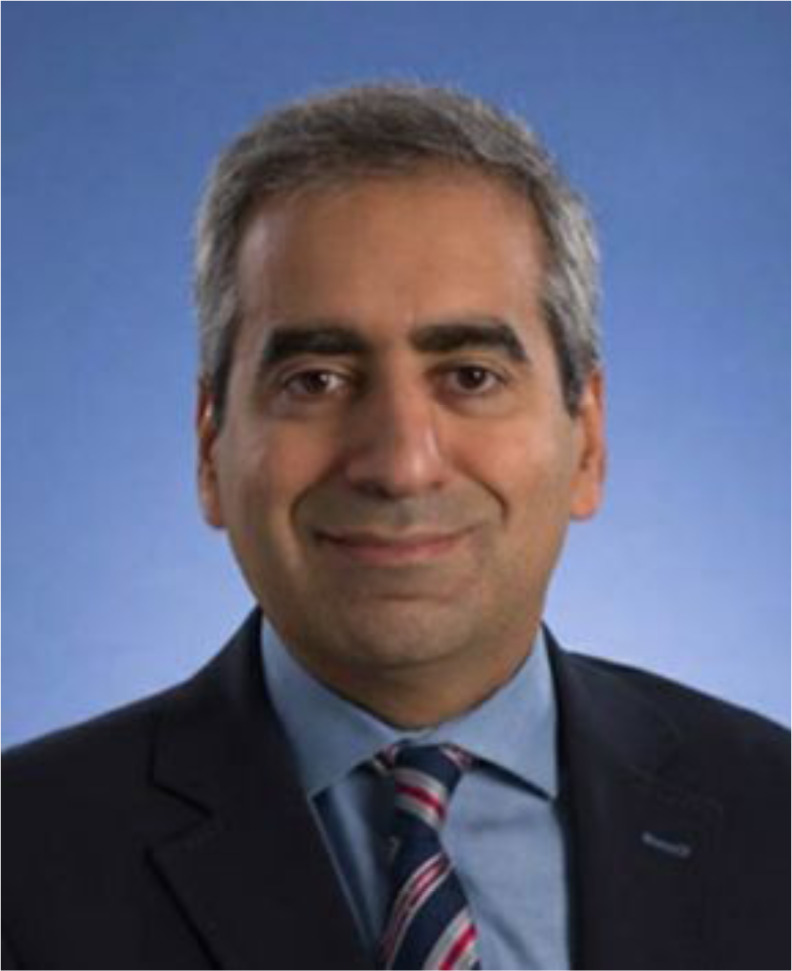
Professor Korosh Khalili, who introduced Radiology fellowship training for the first time in Ethiopia.

**Figure 5:**
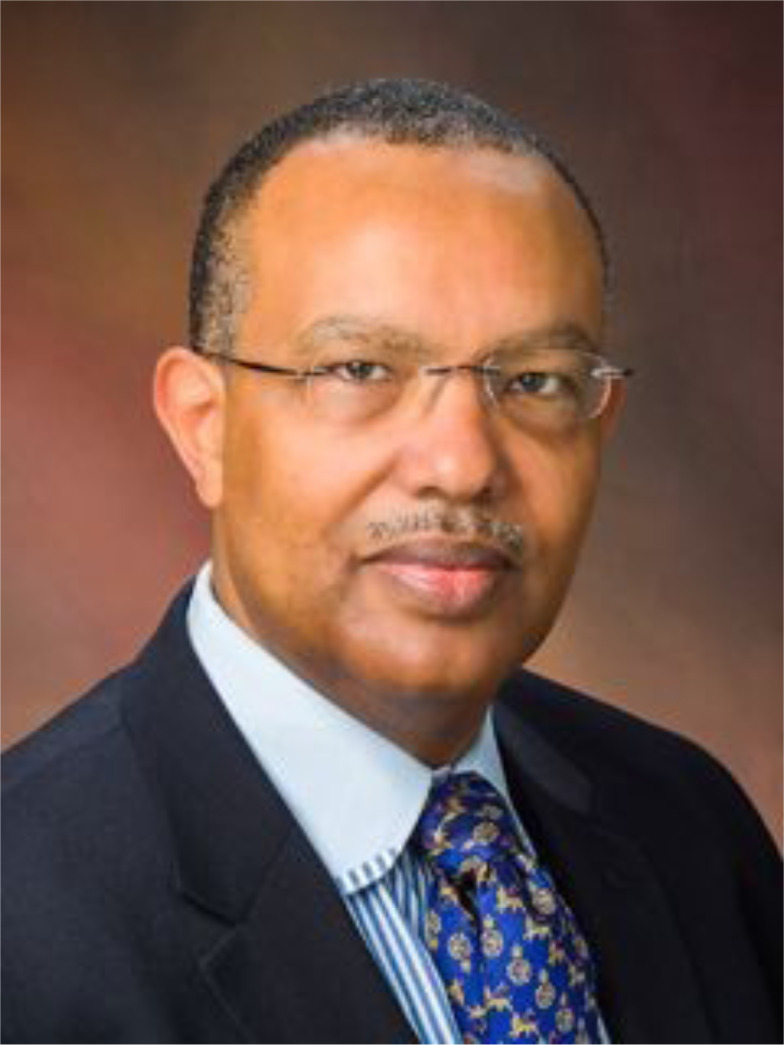
Professor Kassa Darge, who introduced fellowship training in Pediatric Radiology.

1. The Toronto Addis Ababa Academic Collaboration (TAAC) was founded in 2008 to address the capacity building for health professional education and graduate programs. The collaboration initially partnered psychiatry through the Toronto Addis Ababa Psychiatry Program [TAAPP] with the subsequent partnership with other medical disciplines in the university. The collaboration between the Department of Medical Imaging at the University of Toronto and the Department of Radiology at AAU was born in 2012 through which the first radiology subspecialty training was introduced in Body Imaging with the leadership of Professor Korosh Khalili (fig. 4). Moreover, Professor Korosh Khalili initiated and coordinated fellowship training in cardiothoracic and musculoskeletal radiology too.
2. The collaboration between the Children's Hospital of Philadelphia and the Department of Radiology at Addis Ababa university was initially established 14 years back through an Ethiopian Radiologist, Alumni of TASH, practicing at CHOP, Professor Kassa Darge (fig. 5) through the outreach program of CHOP. The collaboration initially was shouldered by a single radiologist, Professor Kasa Darge, which subsequently involved many faculty members of CHOP. The initial objective was to support the residency program at the department of radiology at AAU. Eventually, the pediatric radiology fellowship was born in 2015 with two faculties at AAU joining the program.
3. There were also academic collaborations between the department of radiology at AAU and Emory University through the faculty visitor program which supported the initiation and establishment of the neuroradiology subspecialty program.

The case from SPHMMC

1. The initiation and fostering of the partnership of SPHMMC and Michigan university were done by Professor Senait Fiseha. The collaboration ranged across many residencies and fellowship training that has been undertaken at SPHMMC and abroad including Kidney transplant fellowship training and service. The radiology department was among the many departments which have enjoyed both technical and financial support through the collaboration to launch and develop the first Radiology Residency and Radiology fellowship training.
2. SPHMMC has also enjoyed the partnership with KU at the beginning in the areas of interventional Radiology and Breast and Women's imaging. The partnership has now grown into Radiology Fellowship programs in many radiology subspecialties.
3. Interventional Radiology fellowship training was successfully conducted through collaboration created with the Korean Society of Interventional Radiology (KSIR) and Myungsang Christian Medical Center.

All the residency and fellowship training at SPHMMC was initially shouldered by two of the faculty members namely, Drs. Alemayehu Bedane and Ashenafi Aberra, who created an effective and successful collaboration with the above institutions. Drs. Nebiyu Metaferia and Bethelihem Gelaw from Kansas were instrumental in the collaboration created with the University of Kansas.

Currently, there are close to 40 radiology subspecialists practicing in Ethiopia since its introduction. There has been quite a significant improvement in the quality of the residency training, research output, and tertiary healthcare provision.

## Discussion

The Ethiopian radiology practice showed progress over the last four decades despite the difficulties in addressing the country's need for the profession in terms of number and diversity. In addition to the small number of radiology teaching institutions that cannot address the country's needs, only two of the five radiology teaching institutions are currently providing subspecialty training in different disciplines. Collaboration with international radiology teaching institutions is of crucial importance in the development of radiology practice.

The graduate radiology program, as mentioned above, is firmly established and showing some progress. With regards to service provision, the same cannot be said for public hospitals where scale-up in radiology has not reached the desired level on a par with professional manpower production. A local study on radiology service in Addis Ababa public hospitals published in 2011(5), noted poor radiological infrastructure, poor level of support, and the basic nature of the radiological service in the capital where 25% of the equipment were not functional at the time of the study and 98% of the service were limited to plain x-ray and ultrasound (7). Not much has changed since that time in our view. Most of the newly added cross-sectional modalities are not functional and are given minimal attention, which in the author's opinion is a bottleneck for the expansion of radiology services in the country at large. The scenario in the private sector is much better and high-end imaging equipment and infrastructure is, to a degree, available and managed better in comparison, but nonetheless, there is no well-established public-private partnership to exploit the opportunity and fill the gap in terms of new radiology technologies. Lack of commitment by the public authorities and affordability of radiology examinations in the private setting are still the issue that has to be addressed for better public-private partnerships to develop, which in turn supports radiology expansion (both radiology education and service).

Even if post-graduate expansion was launched by the higher education authority in 2003, there has been no significant change in the number of radiology graduates thereafter since the expansion happened in the only radiology teaching institute at AAU. The institution was already overstretched with the existing constraint of human and material resources to increase its intake capacity in line with the country's needs. As shown in figure 3, there was a steep increase in the number of radiologists with the introduction of new radiology training institutes after the year 2017. Therefore, expanding radiology training by recruiting new radiology training sites in the different regions of the country should be given emphasis rather than increasing the intake capacity of the already existing institutes to rapidly increase trained professionals.

The status of global radiology- is such that it is expanding and developing by leaps and bounds, and has reached an age where the use of digital technology and artificial intelligence have now taken a firm root in the imaging practice, especially in the developed world. There is still a lot to be desired concerning our situation due to the critical shortage of professionals and modern technology, favorable policy, and strategy for the development of radiology practice, on the other hand, International collaborations with institutions from developed nations are vital in keeping the track of international radiology development and proved to be an effective method in developing the radiology practice in resource-poor settings like Ethiopia.

The radiology practice in Ethiopia is expanding and the introduction of subspeciality training is fruitful. The authors recommend additional efforts to be taken by health authorities and all relevant stakeholders to expand the radiology training in Ethiopia to meet the country's needs. Public-private partnerships and collaborations with international institutions should be emphasized to alleviate the shortage of resources, both professional and financial.
